# Speed of Processing (SoP) Training Plus α-tACS in People With Mild Cognitive Impairment: A Double Blind, Parallel, Placebo Controlled Trial Study Protocol

**DOI:** 10.3389/fnagi.2022.880510

**Published:** 2022-07-14

**Authors:** Jorge Leite, Óscar F. Gonçalves, Sandra Carvalho

**Affiliations:** ^1^Portucalense Institute for Human Development—INPP, Portucalense University, Porto, Portugal; ^2^Portuguese Network for the Psychological Neuroscience, Portugal; ^3^Proaction Laboratory, CINEICC, Faculty of Psychology and Educational Sciences, University of Coimbra, Coimbra, Portugal; ^4^Department of Education and Psychology and William James Center for Research, University of Aveiro, Campus Universitário de Santiago, Aveiro, Portugal; ^5^The Psychology Research Centre (CIPsi), School of Psychology, University of Minho, Braga, Portugal

**Keywords:** tACS (transcranial alternating current stimulation), cognitive training, MCI (mild cognitive impairment), NIBS and cognition, EEG

## Abstract

Several cognitive training programs, alone or in combination with non-invasive brain stimulation have been tested in order to ameliorate age-related cognitive impairments, such as the ones found in Mild Cognitive Impairment (MCI). However, the effects of Cognitive Training (CT)—combined or not—with several forms of non-invasive brain stimulation have been modest at most. We aim to assess if Speed of Processing (SoP) training combined with alpha transcranial alternating current stimulation (α-tACS) is able to increase speed of processing as assessed by the Useful Field of View (UFOV), when comparing to SoP training or active α-tACS alone. Moreover, we want to assess if those changes in speed of processing transfer to other cognitive domains, such as memory, language and executive functioning by using the NIH EXAMINER. We also want to test the mechanisms underlying these interventions, namely brain connectivity and coherence as assessed by electroencephalography (EEG). To that purpose, our proposal is to enroll 327 elders diagnosed with MCI in a double-blinded, parallel randomized clinical trial assessing the effects of combining SoP with alpha endogenous tACS (either active or sham) in people with MCI. Participants will perform an intervention that will last for 15 sessions. For the first 3 weeks, participants will receive nine sessions of the intervention, and then will receive two sessions per week (i.e., booster) for the following 3 weeks. They will then be assessed at 1, 3, and 6 months after the intervention has ended. This will allow us to detect the immediate, and long-term effects of the interventions, as well as to probe the mechanisms underlying its effects.

**Clinical Trial Registration:** Clinicaltrials.gov, Identifier: NCT05198726.

## Introduction

The major predictor for cognitive decline is age (Tilvis et al., 2004; Schaeverbeke et al., 2021). Despite heterogenous profiles in terms of progression and severity, a dysexecutive syndrome that can be detected in early onset variants of Alzheimer’s disease (Townley et al., 2020), or in mild cognitive impairment (i.e., MCI; Blanco Martín et al., 2016) has been commonly reported. This dysexecutive syndrome translates to difficulties in terms of crystalized and fluid intelligence, processing speed, attention, memory, language, and executive functioning (Harada et al., 2013), and as such, constitute potential targets for cognitive training (CT) in these populations.

In the absence of official number for the lifetime prevalence of MCI, a recent meta-analysis suggested that MCI can affect from 22.5 (aged 75–79) to 60.1 persons (aged 85 years or older) per 1,000 persons-year (Gillis et al., 2019). Moreover, several studies suggested that people with MCI are more prone to develop dementia (Baker et al., 2008), especially if the degree of functional baseline is accounted for (Farias et al., 2009). However, there are also some reports that people with MCI reverted their cognitive condition to a normal stage (Hu et al., 2020). This is especially important because, this may end up lowering the risk of developing dementia for some people with MCI. Furthermore, even with the recent advances in terms of medication, especially the aducanumab, its impact on cognition is still a matter of intense debate (Walsh et al., 2021).

In that sense, several interventions under the general umbrella of CT have been extensively studied in order to test if CT, will be able to minimize or revert the effects of age-related cognitive decline in older adults (Irazoki et al., 2020 for review; Willis et al., 2006). For instance, a program using cognitive training in people with MCI that consisted of 60-min sessions, 3 times a week, during 6 months was able to induce a medium size effect improvement in the Alzheimer’s Disease Assessment Scale-Cognitive Subscale (ADAS-Cog; Train the Brain Consortium, 2017). Moreover, previous meta-analyses suggested that cognitive training in healthy volunteers can indeed increase fluid intelligence (Karbach and Verhaeghen, 2014; Au et al., 2015). However, other meta-analysis suggested that the effects of CT in the elderly are modest at most (Teixeira-Santos et al., 2019).

Interestingly enough, a recent randomized clinical trial (RCT) with 10 years of follow-up, compared the efficacy of three types of cognitive training in 2,802 older adults. In this trial, participants received either Working memory, Reasoning or Speed of Processing training compared to a waiting list. The CT consisted of 10 sessions during six weeks, and then up to four booster sessions at the 11th month, and finally another up to four booster sessions at the 35th month. Working memory or reasoning training did not reduce the risk of developing dementia [43% met the diagnosis of dementia criterion, 28% met the Mini Mental State Examination (MMSE) criterion, 12% met the psychometric criterion; 15% and 2% met all three criteria] when compared to controls, but that was not the case for speed of processing (SoP) training (Edwards et al., 2017). In another study, SoP training increased participant’s instrumental everyday abilities, including driving abilities (Ball et al., 2007). Moreover, transfer effects to other cognitive domains (e.g., attention, memory) and increases in connectivity within the brain’s central executive network following SoP training were also reported (Lin et al., 2016). This is not surprising, as SoP has been shown to be an important marker for individual differences in terms of structural connectivity (Bennett and Madden, 2014). Moreover, higher order cognitive functions, such as executive functioning, rely on efficient communication between brain regions, and as such, decreases in SoP are a strong predictor of age-related decline in several cognitive domains (Gao et al., 2020). This reflects a connection deterioration between distal brain regions in what has been called the “disconnected brain” framework (Fjell et al., 2013). Thus, an increase of target engagement may be possible by tuning-in the neural population towards the trained ability, by using non-invasive brain stimulation (NIBS).

We have previously shown that we were able to modulate cognition using transcranial direct current stimulation (tDCS), namely on executive functioning (Leite et al., 2011, 2013), working memory (Carvalho et al., 2015a), empathy (Rêgo et al., 2015), approach/avoidance motivation underlying craving (Carvalho et al., 2018), attention and pain (Silva et al., 2017; Santos et al., 2018). Furthermore, by understanding the underlying mechanisms of the intervention in the brain, it is possible to develop neural based interventions, whose ultimate aim is to optimize the target engagement (Carvalho et al., 2015b).

One possible way of doing so, is to combine SoP training with another technique— transcranial alternating current stimulation (tACS) —which has been shown to entrain brain activity (Antal and Herrmann, 2016). Entrainment refers to the frequency dependent response of brain waves in order to match the frequency of the stimulus that are being applied to the brain (Huang and Charyton, 2008). Alpha entrainment may be of particular interest for SoP. For instance, a study has already showed that speed of processing is associated with changes in this specific band (Hilla et al., 2020). By combining the two interventions, it will be hypothetically possible to optimize brain communication between distal regions, and thus, increase cognitive performance. According to the Communication through Coherence hypothesis (CTC), effective connectivity requires rhythmic synchronization between pre and post synaptic neurons and their coherence. Thus, in the absence of coherence, inputs arrive at random phases of the excitability cycle, which result in lower connectivity (and efficiency; Fries, 2015). Therefore, coherence between pre and postsynaptic neurons is required for efficient communication. In the case of absence of coherence (as in the case of a “disconnected brain due to ageing), performance is impaired.

In this sense, alpha tACS (α-tACS) may be a suitable combination for SoP training. For instance, α-tACS induces preferential phase synchronization of fast spiking neurons, thus resulting in an entrainment process of alpha oscillations (Huang et al., 2021). Moreover, alpha band is thought to work as a brain timer, setting up excitation and/or inhibition microstates, which will optimize incoming information processing or inhibition (Mathewson et al., 2011). Furthermore, α-tACS is able to modulate temporal resolution of human vision by acting over the segregation/integration time window of visual information, thus inducing participants to integrate more often two stimuli into one percept (Battaglini et al., 2020); or even to modulate information transfer in the premotor-cerebellar loop during learning (Schubert et al., 2021).

Interestingly enough, alpha activity seems to be one of the most distinguishable alterations in the human brain due ageing, with several studies showing changes in terms of alpha power, coherence and peak frequency in older adults, when comparing to younger ones (Purdon et al., 2015; Beese et al., 2019; Elshafei et al., 2020).

Here the goal is to use each participant’s endogenous peak alpha frequency to guide endogenous alpha phase-tACS (α-tACS) to further increase brain connectivity within brain networks (Bächinger et al., 2017).

In this sense, the current proposal aims to combine two interventions that have already shown promising results: a specific SoP training that has been shown to produce results in the elderly (Edwards et al., 2017) and an intervention that has been shown to entrain brain activity (Huang et al., 2021), as a means of increasing brain connectivity (Bächinger et al., 2017).

## Methods

### Purpose, Primary, and Secondary Outcomes

#### Primary Outcomes

The primary outcome will be changes from baseline in the *Useful Field of View* (*UFOV*) Assessment at week 3. UFOV is a good indicator of visual processing speed (Ball et al., 1988). The endpoint at week 3 was chosen because it is at the end of the intensive phase, and will be complemented by a co-primary endpoint at week 6 (after booster sessions). Long-term effects will be assessed at 1, 3, and 6 months after the stimulation has ended.

#### Secondary Outcomes

##### NIH Executive Abilities: Measures and Instruments for Neurobehavioral Evaluation and Research (NIH EXAMINER)

Aims at providing a robust measure of executive functioning, namely in terms of working memory, inhibition, set shifting, fluency, insight, planning, social cognition, and behavior (Kramer et al., 2014). Moreover, it seems to be sensitive enough to detect cognitive changes even in pre-symptomatic frontotemporal lobar degeneration (Staffaroni et al., 2020). It will be used to assess for potential transfer effects from speed of processing towards several core skills underlying executive function.

##### Electroencephalography (EEG) Data

EEG can be used as an outcome predictor for the intervention, or prospectively in order to determine the chance of progressing towards MCI or other Dementia-like state (Poil et al., 2013). Moreover, EEG can be used to assess changes in brain connectivity in people with MCI (Tóth et al., 2014).

### Trial Design, Setting, and Registration

**Figure 1:**
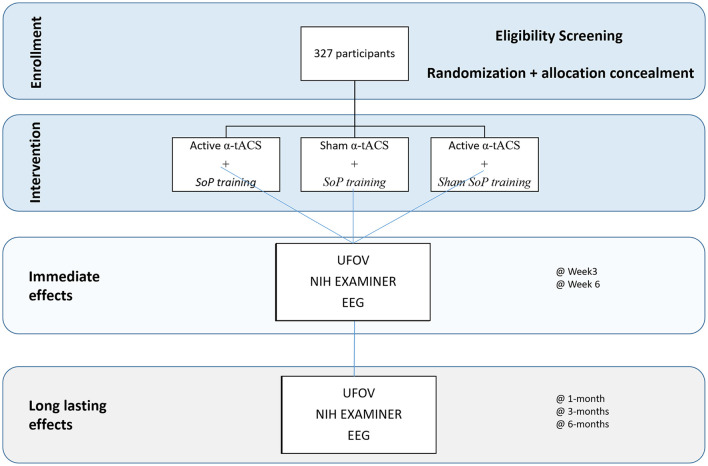
Schematic representation of the protocol.

The trial will be an exploratory three arm parallel group, double blinded controlled trial, with an 1:1 allocation (see Figure 1). Data will be collected from clinical outpatient services from the Northern and Center Regions of Portugal. This trial has been registered at Clinicaltrials.gov with the identifier: NCT05198726.

Participants will perform a pre-screening procedure in order to assess their eligibility for the study. If they are eligible, they will be screened by a trained clinician (neurologist or neuropsychologist) in order to assess if there is evidence of modest cognitive decline from a previous level of performance; that these deficits do not impair participant’s independence; that the cognitive decline is not a result of a delirium, or better explained by other mental disorder such as depression or schizophrenia as suggested by DSM-V (American Psychiatric Association, 2013). Participants that screen in, will perform a baseline assessment, which will consist of the UFOV, the NIH EXAMINER, and the EEG data. Then they will perform an intervention that will last for 15 sessions. For the first 3 weeks, participants will receive nine sessions of the intervention, and then will receive two sessions per week (i.e., booster) for the following 3 weeks. We will then conduct three follow-up assessments at 1, 3, and 6 months after the stimulation has ended. Assessments will be similar across all study points.

### Participants

Participants will be community-dwelling adults aged 65 years and older, with cognitive complains in memory or other functions, which have declined over time. Participants will be excluded if they are less than 65, score in the mini mental examination less than 23 (or over 28), diagnosis of Alzheimer’s Disease, any other medical condition that may predispose participants for a forthcoming decline in function, or that may increase their risk of mortality during the duration of the study. Participants will also be excluded if they have any contra-indication to tACS, severe sensory losses or communication difficulties that will prevent them from successfully complete the training or had any cognitive training in the past.

Voluntary and written informed consent will be obtained from the participants by a researcher, which will explain in detail the procedures involved in the study.

### Description of the Interventions

#### Speed of Processing (SoP)

**Figure 2:**
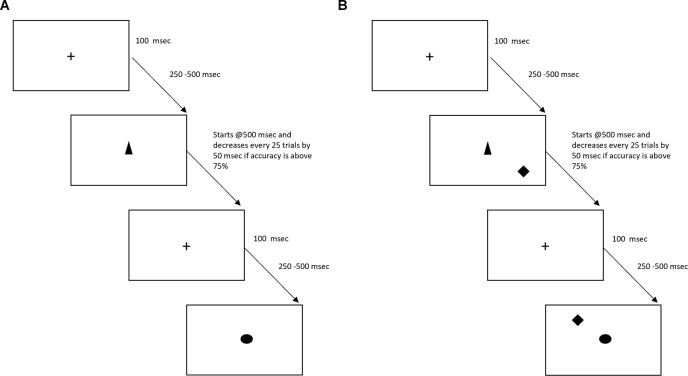
Schematic representation of the two first conditions of the Speed of Processing (SoP) task. Panel **(A)** represents condition 1, with only one target. Panel **(B)** represents condition 2, in which a second target is added (i.e., diamond).

There will be a total of 15 sessions (30-min per session) of SoP training. These sessions will be performed three times a week for the first 3 weeks and two times a week for the following 3 weeks, and will consist of four conditions. In condition 1, participants will be asked to identify objects at increasingly brief exposures (see Figure 2A). For condition 2, identification of stimulus on the center of the screen will be requested from participants, while a second stimulus will appear somewhere in peripheral vision—stimulus duration, difficulty of the central localization task or the area in which targets may be located, will be used to change the level of difficulty (see Figure 2B). Condition 3 is similar to condition 2, but visual distractors are added. Finally, for condition 4, an auditory identification component will be superimposed over the visual task (Jobe et al., 2001).

Objects time exposure will decrease by 50 ms if in the preceding block of 20 trials, participants successfully recognized the object in at least 75% of trials. If not, time exposure of objects will go up by 50 ms. Once exposure of objects cannot be reduced for three consecutive blocks of 20 trials, participants may proceed to the next condition. Visual targets will be approximately 2 cm in wide and height, which represents a visual angle of 1.9° at a 60 cm distance. For the sham version of the task, objects time exposure will be fixed across all trials (i.e., 500 ms).

#### Endogenous Alpha-Transcranial Alternating Current Stimulation (α-tACS)

Participants will do a run-in phase (EEG without α-tACS) during the first 5-min of each training session in order to trace the individual alpha frequency (IAF) over occipital regions (defined as the maximum alpha value within the 7–13 Hz range), which will then be used to deliver the 2 mA α-tACS (based on the IAF) over the prefrontal cortex (PFC; i.e., F3 and F4) for 20-min, using 35 cm^2^ sponges. The PFC was chosen due to his age-related decline (Fjell et al., 2013) and to his involvement in visual target processing (Madden et al., 2004). For the sham tACS, a 45-s of active α-tACS will be applied. This will allow to mimic the initial sensation of tACS. The computer screen will randomly flicker across the experiment for the sham tACS, because active tACS bellow 40 Hz is likely accompanied by visual flicker sensations. Thus, we plan to use the computer screen to mimic this flickering for the sham tACS condition. In order to continuously monitor electroencephalographic (EEG) activity and deliver α-tACS during task performance, a custom Loop-IT solution from Neurocare (Germany) will be used.

### Outcomes

Main outcome will be the Useful Field of View (UFOV) test for speed of processing and attention (Ball et al., 1988). The UFOV is a test that was designed to evaluate daily life difficulties experienced by the elderly, such tasks that require divided visual attention and have to be undertaken rapidly (speed of processing). The four-subtest stimulus will be used for the computerized version, namely identification alone; divided attention; selective attention; selective attention in conjunction with same/different discriminations will be used (Edwards et al., 2006). UFOV is expected to be completed in 10 min and participants are asked to touch the screen as soon as they identify a target (i.e., a car silhouette inside a box), with the target exposure ranging from 16.67 to 500 ms. In order to probe divided attention, a peripheral target is added to the central target. For the selective attention, distractors are added; while for the more demanding version, there are two central targets, and participant are asked to make a judgment if they are similar or not.

The NIH EXAMINER battery will be used for assessing transfer effects of the intervention to untrained domains. The NIH EXAMINER comprises cognitive control, verbal fluency, and working memory assessments (Possin et al., 2013). Namely, NIH EXAMINER is a computerized version of several tasks targeting working memory, inhibition, set shifting, fluency, insight, planning, social cognition, and behavior, which allows for the calculation of a composite score (Kramer et al., 2014). As the time to complete several tasks is performance dependent, we expect participants to complete the NIH EXAMINER between 30 and 60 min.

Resting state EEG will be used as a surrogate outcome, and will be performed before and immediately after the intervention in order to assess power spectral density changes, cross frequency couplings and coherence among electrodes. Participants will also perform a short auditory oddball task (Fz, Cz, Pz, and Oz) because the P3 wave is a good marker of brain’s speed of processing. Analysis will be considered exploratory if the dropout rate reaches 20%.

At week 3 and 6 outcomes will be collected the following business day, due to particular characteristics of the sample.

### Sample Size

No previous studies using tACS and SoP have used UFOV as a primary outcome, and as such, we are using the UFOV test-retest data from Bentley et al. (2012). For older adults the mean (SD) test-retest difference (i.e., consistency between measurements at two different timepoints on the same sample) between UFOV applications was 56.2 (74.8). In order to show an effect, our intervention should at least surpass the mean test-retest difference. So we ran three different scenarios: 0.50, 0.75, and 1.00 SD below mean difference for older adults, with a power of 0.8 and alpha of 0.05. In these scenarios, a minimum of 17 participants and a maximum of 64 per group would be required in order to detect a Cohen’s d of 1 and 0.5, respectively. However, these values were above the mean test-retest difference for younger adults, namely 31.5 (43.7). As such we used this value as our cut off point, and with a power of 0.8 and alpha of 0.05 we would need at least 98 participants per group. If an attrition rate of 10% is considered, 109 participants per group will be required. However, having trial feasibility into consideration, we decided to conduct a interim analysis for futility as soon as 19 participants per group are reached, then at 32 participants per group and finally at 71 participants per group (always considering the 10% attrition rate).

### Recruitment

Recruitment will be made in the north and center regions of Portugal, using broad community advertisement strategies (i.e., Facebook page, flyers) and by targeting specific organizations that usually work with the elderly such as senior universities and assisted care home facilities.

### Randomization

Participants will be block randomized (random block sizes of 2, 4 or 6) by a computerized software to one of the interventions. Allocation concealment will be kept using a method of closed envelops, which will only designate Group A or B. A or B will then be used to set up a masked study mode on the device. Randomization list will be kept on a secured storage, and the access will be restricted to the Principal Investigator. Enrolment of participants will be performed by a researcher of the trial, and the allocation will be performed immediately prior to the beginning of the stimulation sessions.

### Blinding Procedures

This will be a double blind design, in which trial participants, researchers providing the interventions and outcome assessors will be blinded to the intervention. This will be achieved using study mode of the devices and employing sham procedures for the α-tACS. Participants will be unblinded if they experience any unexpected adverse effects. At the end of their participation, blinding assessment will be performed to both participants and researchers who assessed the outcomes, namely by asking about the stimulation that they thought they had received (or applied) and to rate how confident they are in their guesses.

### Assessments

#### Useful Field of View (UFOV)

UFOV is a very well validated instrument to assess speed of processing and will be administered before the intervention and at the end of week 3 (see Figures 1, 2). This time point was chosen in order to minimize loss to follow-up. As there is some evidence that there may be a ceiling effect of CT (which may be the case for SoP also), we will also collect a co-primary endpoint at the end of the training (i.e., week 6; see Figures 1, 2).

#### NIH EXAMINER

The NIH Executive Abilities: Measures and Instruments for Neurobehavioral Evaluation and Research-EXAMINER battery will be used to assess transfer effects to untrained domains, such as executive function, insight or social cognition and behavior (Kramer et al., 2014). The NIH EXAMINER was previously developed to be used as a trial endpoint, and is organized into subtests targeting working memory, inhibition, set shifting fluency, insight, planning, social cognition and behavior, that allows us to obtain an executive composite score for each individual.

#### EEG Data

EEG data will be acquired using a 8-channel Loop-IT (Neurocare, Ilmenau, Germany) following the 10/20 system, in a continuous mode at a digitization rate of 500 Hz, with a bandpass filter of 0.01–100 Hz. The EOG will be recorded from two additional bipolar channels. Electrode impedances will be kept below 5 kΩ. We will be especially interested in changes in α- power, P300 from the oddball task, Event Related Desynchronization (ERD; for alpha attenuation) and Phase amplitude coupling (PAC; mainly α−γ).

#### MOCA, MMSE, and GDS

Participants will be screened using the Montreal Cognitive Assessment (MOCA; Nasreddine et al., 2005) and the Mini-mental State Examination (MMSE; Folstein et al., 1975). The Geriatric Depression Scale (GDS) short version (Yesavage and Sheikh, 1986) to track depression levels that could be used as a covariate in the analysis.

### Data Management and Access to Data

Collected data will be handled and stored accordingly to national and international regulations, always respecting the best (and stringent) practices available. All personal data will be de-identified and coded. The code will be stored in a locked storage with restricted access. Digital data will be stored using REDCap. A secure encrypted OneDrive will be used for the EEG data. We will use EEGLab, Matlab and SPSS version 28 to perform the data analysis. All further processing of data will follow an ethics committee approval and the data used will remain de-identified and coded, following the data-sharing agreement.

### Statistical Analysis

#### Primary Outcome

We expect that all groups, after the intervention, have increased performance in the UFOV scores, however we also expect that the group with the combined active α-tACS and SoP training would present the largest increase in scores, when comparing to either intervention alone. This will be tested by calculating the delta (week 3 or 6—baseline), and by performing independent one-way ANOVAS with intervention as a factor. Moreover, with the present design, we can track the intervention effects in several time points, namely between baseline, at week 3 and 6 of the interventions. This will be performed by means of an exploratory mixed model-ANOVAS, with time as within subject factor and intervention as between subject factor. As we cannot control at this point for several variables such as gender, depression or years of education, if there are significant differences between groups after randomization, we will include those covariates in the analysis, performing ANCOVAS instead of ANOVAS.

#### Secondary Outcomes

##### NIH Examiner

The NIH EXAMINER battery is important to address transfer effects to untrained domains, such as executive function, insight or social cognition and behavior. Chi-square tests will be used for week 3 and 6 reliable change indexes. Reliable Change Index (RCI) will be calculated by using the standard deviation of the reference data-set, and the test-retest reliability (Cronbach’s alpha). As we want the RCI, the difference between measurements will be compared to the S_Diff_ multiplied by 1.96 (Jacobson and Truax, 1991). This will allow for a categorial YES/NO variable for the chi-square tests. Moreover, with the present design, we can track the intervention effects in several time points, namely at baseline, week 3 and 6 of the treatment. This will be performed by means of chi-squares, with time as within subject factor and intervention as between subject factor.

##### EEG Data

**Figure 3:**
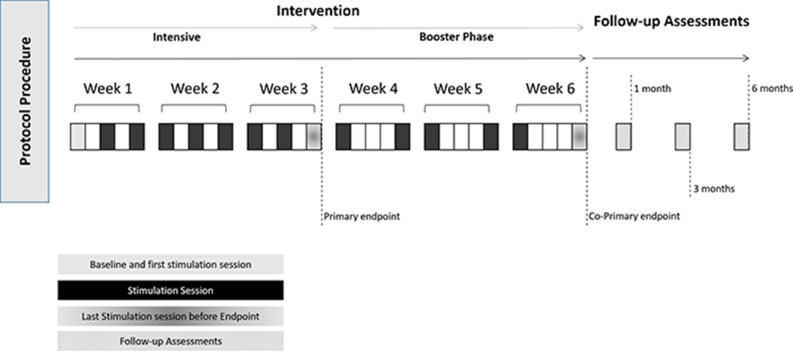
Schematic representation of the overall procedure, including session procedure. Follow-up assessments will start 1 month after the intervention has ended.

EEGLab^1^ will be used for EEG data pre-processing and analysis. Independent Component Analysis (ICA) will be used for artifact rejection and IAF. IAF will be determined in the first 5 min of a 10-min run in phase in which participant will not receive any stimulation, with alternating cycles between eyes open and eyes closed. Fast Fourier Transformation (FFT) will be applied to posterior α. Power estimates will be obtained by squaring filtered EEG signals and then band power values will be averaged for both the pre-stimulus reference period and the task intervals. We will then segment the trials in blocks of 5-s, and average the brain activity for those blocks. In order to prevent the mismatch between α-tACS and measured α, α peak will be measured always during the first 5-min of the session (without training) and in the 5-min immediately after stimulation has ended (see Figure 3). Source information toolbox^2^ will be used for the coherence and connectivity analysis between the eight electrodes (FP1, FP2, C3, C4, T3, T4, O1, and O2) without pre-specifying specific Region of Interest (ROI) comparisons.

##### Long-Term Effects

Assessments at 3 and 6 months of follow-up will be critical for understanding the longterm effects of the intervention. We will use the reliable change index (RCI; Ferguson et al., 2002) for the primary (i.e., UFOV) and secondary endpoints such as the NIH EXAMINER. By assessing if the changes in test scores are reliable across those time points, our understanding about the long lasting effects of the intervention will improve, while potential placebo effects that are not expected to persist for that long will be mitigated. This will be performed by means of Chi-squares, with time as within subject factor and intervention as between subject factor. At the 1, 3, and 6 month follow-ups, participants will also perform the EEG, in order to allow for the correlation between changes in α- power, Event Related Desynchronization (ERD; for alpha attenuation), Phase amplitude coupling (PAC; mainly α−γ) and with the reliable change indexes (RCI) from the primary (UFOV) and secondary outcomes (NIH EXAMINER), which will increase our understanding about the mechanisms underlying this intervention.

### Ethics and Dissemination

The trial is currently undergoing clinical trials registry and is approved by the local ethics committee—Comissão de Ética para a Saúde–CES02/21.

### Confidentiality

Data collection forms will be de-identified and participants will be given codes, composed by letters and numbers to which all data will be linked. File records containing personal data will be stored in a file cabinet room, with restricted access. Electronic data will be kept in secure local servers during the period of the study and up to 5 years after the study has ended. Sensitive data will be handled and stored according to national and European GDPR regulations.

### Dissemination Policy

Deidentified and coded data will be available to researchers upon request. Results of the study will be presented in scientific meetings, as well as in sessions to the general public. Results of this study will be published in peer reviewed journals.

## Discussion

The current work describes a protocol for a parallel, randomized, double-blind sham controlled trial to test the effects of the combination of active α-tACS with Speed of Processing (SoP) training, in trained (as measured by the UFOV) and untrained domains (as measured by the NIH EXAMINER), in people with Mild Cognitive impairment (MCI). Assessing trained transfer effects to other cognitive domains (and even to daily life activities) are extremely important, as the current literature is full of mixed results concerning the transfer effects of cognitive training (Sala and Gobet, 2017).

With the present design we expect that all groups will improve, however we expect that the combined intervention will be better at target engagement, and as such, participants on that specific arm, will improve more than the others. So what we aim to address with the current protocol is if the combination of SoP and active α-tACS is better than any intervention alone. It is important to note, that the current design is not a full factorial (i.e., there is no placebo intervention alone). This was a choice, as an inclusion of another group would increase the costs of trial, jeopardizing its feasibility.

Moreover, the current protocol also aims at studying the underlying mechanisms of the intervention. By studying the mechanisms underlying the interventions, it is possible to understand the role of the underlying neural circuitry (Gonçalves et al., 2016) as well as to detect specific candidates for brain biomarkers, which could be used to guide interventions, or to serve as outcome predictor (Yavari et al., 2016; Ovadia-Caro et al., 2019).

For instance, by using electrophysiological measures, during baseline and post-intervention, it will be possible to probe for connectivity changes within the brain, especially in the resting state networks. Moreover, it will be possible to unravel how those changes in connectivity relate to cognitive performance within the “disconnected brain” framework (Fjell et al., 2013, 2016). Furthermore, it will be possible to study potential phase amplitude coupling (PAC) between the alpha and other EEG bands, such as the gamma (γ; Canolty and Knight, 2010), which is thought to be responsible for memory and attentional processes (Herrmann and Mecklinger, 2001). By understanding such changes in the brain, the required algorithms for EEG guided stimulation closed-loops can be further developed (Leite et al., 2017).

Another important aspect of this study protocol is the understanding of the long-term effects of the intervention. The first follow-up assessment will occur at 1 month after the intervention has ended, and will be repeated at 3 and 6 months (see Figure 2). These long-term assessments are extremely important, as there is substantial evidence for placebo effects in healthy volunteers due to CT. In a recent study, participants that responded to a catchy flyer, suggesting that they were going to undertake a 1 h CT that would improve their cognitive performance and their fluid intelligence, improved between 5 and 10 points in a standard IQ scale; while those that responded to a nonsuggestive flyer (i.e., participating in a 1 h session in exchange for course credits) did not (Foroughi et al., 2016). This study highlights the importance of accounting for placebo effects when assessing the effects of CT. Despite the fact that there is no clear framework for estimating the duration of placebo effects, there are suggestions that the placebo effects may last up to 6 or 12 months (Hansen et al., 1996; Previtali et al., 2021). We will be using reliable change index (RCI) in order to assess if the changes in participant’s performance across time are reliable or not (Barker-Collo and Purdy, 2013). By using multiple assessments and especially the RCI we expect to mitigate any possible confounders arising from placebo effects.

The main challenge will be the loss to follow-up. Nonetheless, the team will focus on developing strategies to minimize this, such as sending reminders (phone, letter or email), by involving caregivers in the process, among others. If participants are not able (or willing to perform the EEG during follow-up), they will be offered the opportunity to perform the remaining follow-up assessments remotely, using videoconference systems, by phone or by the means of server based cognitive tasks.

This proposal has some ethical concerns that need to be addressed according to national and international regulations. The participation of subjects will always be informed, consented and voluntary. If participants are vulnerable individuals or groups then research activities will only be performed if all potential risks are minimized, and the outcome of research is valuable enough that can have an impact in their quality of life, mental health or well-being. The current proposal involves the use of physical techniques, which are not invasive and do not represent more than minimal risk.

Therefore, the current proposal aims at combining speed of processing training (SoP) with endogenous alpha tACS, by developing an EEG-tACS closed loop solution. Additionally, we plan to use executive measures to assess if speed of processing training transfers to other higher order cognitive domains. Furthermore, we want to explore how changes in cognition are related to changes in the brain, as assessed by the EEG, namely connectivity across proximal and distal regions, and coupling with other frequencies of interest, such as the alpha-gamma amplitude coupling. In sum, the results of this project will further provide important insights into the mechanisms underlying EEG-tACS closed-loop coupled with cognitive training to enhance Speed of Processing in people with MCI. This proposal will allow us to test a new intervention, at the same time that we explore the mechanism by which the intervention works, and to probe for potential biomarkers that may be useful to guide future interventions.

## Author Contributions

JL and SC designed the protocol. JL drafted the initial version of the manuscript. SC and ÓG contributed to the final version of the manuscript. All authors contributed to the article and approved the submitted version.

## Funding

This work was supported by National Funds through the Portuguese Foundation for Science and Technology (FCT) and co-funded through COMPETE 2020—PO Competitividade e Internacionalização/Portugal 2020/União Europeia, FEDER (Fundos Europeus Estruturais e de Investimento—FEEI) under the number: PTDC/PSI-ESP/30280/2017.

## Conflict of Interest

The authors declare that the research was conducted in the absence of any commercial or financial relationships that could be construed as a potential conflict of interest.

## Publisher’s Note

All claims expressed in this article are solely those of the authors and do not necessarily represent those of their affiliated organizations, or those of the publisher, the editors and the reviewers. Any product that may be evaluated in this article, or claim that may be made by its manufacturer, is not guaranteed or endorsed by the publisher.

## Footnotes

^1^ https://eeglab.org/

^2^ https://sccn.ucsd.edu/wiki/SIFT
